# Vision testing to prevent road traffic accidents in Kenya

**Published:** 2015

**Authors:** Michael Mbee Gichangi, Gladwell Koku Gathecha, Duncan Kibogong

**Affiliations:** Head: Ophthalmic Services Unit, Ministry of Health, Nairobi, Kenya. Email: gichangi58@yahoo.com; Head: Violence and Injury prevention Unit, Division of Non Communicable Diseases, Ministry of Health, Nairobi, Kenya.; Deputy Director: Safety Strategies and Country Committees, National Transport and Safety Authority, Nairobi, Kenya.

Only 28 countries worldwide have laws to address risk factors and prevent road traffic accidents and injuries.^1^ Prevention of road traffic accidents requires guidelines on visual acuity requirements, a standardised examination process and, particularly for commercial drivers, regular enforcement of the visual acuity requirements. Enforcement remains a major challenge, and cannot be done without legislation. The process of legislation has to involve multi-sectoral stakeholders as well as public agreement.

In Kenya, road traffic accidents account for 59.6 injuries and 28.2 deaths per 100,000 population.^2^,^3^ A study involving vision assessment of public service drivers in Nairobi in 2001^4^ found that a significant proportion of drivers who have had an accident also had cataracts. Because driving is a source of income, it is likely that many people might continue driving despite experiencing visual difficulties – perhaps fearing that their income will be at risk if they are identified as having a visual complaint.

In response to the high incidence of accidents in Kenya, Kenya's Traffic Act (revised in 2012) requires that every driver of a public service or commercial vehicle be physically fit (including eyesight and hearing ability) before the license is renewed. Having a mandatory medical assessment before the renewal of a driving license (every 3 years) will also be in line with Kenya's new health policy framework (2014–2030).

## Developing the guidelines

With technical support from the World Health Organization (WHO), a technical working group (TWG) was formed to develop practical guidelines to implement these legal requirements. The group was led by the Ministry of Health's Division of Non-Communicable Diseases (NCD) and included representatives of all of the following:

- Medical professionals: both general practitioners and specialists (ear, nose and throat [ENT] and ophthalmic)
- Professional associations and councils
- The National Transport Safety Authority (NTSA)
- The traffic police department
- Owners of public service vehicles (minibuses – also known as ‘matatus’).

During a series of workshops, the group developed feasible technical criteria and a recommended examination process for drivers (involving visual assessment, hearing assessment, and a general health assessment). Here is what was decided in terms of the criteria for visual assessment and the eye examination process.

## Criteria for visual assessment

Vision is one of the most important sources of information to ensure safe driving. It is important to have good visual acuity and to be able to judge distances so drivers can react appropriately. Applicants should meet the following criteria in order to be certified fit to drive (with spectacles or available correction).

- Two normal eyes which are aligned (no strabismus), freely moving and able to identify images as one (no double vision)
- Visual acuity of at least 6/9 in the best eye
- Visual acuity of at least 6/60 in the other eye
- Normal visual fields.

## The eye examination

The eye examination will be carried out by a registered eye health professional, such as an ophthalmologist, ophthalmic clinical officer, cataract surgeon or ophthalmic nurse, at the county referral health facilities or any other NTSA accredited health facilities, and will entail the following.

1. Visual acuity (VA) testing, performed using a standard (Snellen) visual acuity chart, placed 6 metres away in a well-lit room
2. An anatomical eye examination, using a torch and ophthalmoscope or slit lamp
3. A visual field examination using standard methods, but at least by confrontation method
4. Testing of gross ocular motility to rule out diplopia, and fundoscopy for all patients with (or suspected of having) diabetes or glaucoma.

## The road ahead

Finally, before the mandatory assessment is implemented, a wider public discussion is planned and agreement will be sought on guidelines, the assessment form, and the final medical certificate that will be issued before a license can be renewed. The guidelines will be pilot tested before being Gazetted (i.e. become part of Kenya's law) and will be revised based on trends and new evidence.

Note: These guidelines do not apply to private/small cars.
